# Heart fatty acid-binding protein is associated with phosphorylated tau and longitudinal cognitive changes

**DOI:** 10.3389/fnagi.2022.1008780

**Published:** 2022-10-10

**Authors:** Yan Fu, Zuo-Teng Wang, Liang-Yu Huang, Chen-Chen Tan, Xi-Peng Cao, Lan Tan

**Affiliations:** ^1^Department of Neurology, Qingdao Municipal Hospital, Qingdao University, Qingdao, China; ^2^Clinical Research Center, Qingdao Municipal Hospital, Qingdao University, Qingdao, China

**Keywords:** Alzheimer’s disease, HFABP, lipid metabolism, cognition, cerebrospinal fluid

## Abstract

**Background:**

Perturbation of lipid metabolism is associated with Alzheimer’s disease (AD). Heart fatty acid-binding protein (HFABP) is an adipokine playing an important role in lipid metabolism regulation.

**Materials and methods:**

Two datasets separately enrolled 303 and 197 participants. First, we examine the associations of cerebrospinal fluid (CSF) HFABP levels with cognitive measures [including Mini-Mental State Examination (MMSE), Clinical Dementia Rating sum of boxes (CDRSB), and the cognitive section of Alzheimer’s Disease Assessment Scale] and AD biomarkers (CSF amyloid beta and tau levels). Second, we examine the longitudinal associations of baseline CSF HFABP levels and the variability of HFABP with cognitive measures and AD biomarkers. Structural equation models explored the mediation effects of AD pathologies on cognition.

**Results:**

We found a significant relationship between CSF HFABP level and P-tau (dataset 1: β = 2.04, *p* < 0.001; dataset 2: β = 1.51, *p* < 0.001). We found significant associations of CSF HFABP with longitudinal cognitive measures (dataset 1: ADAS13, β = 0.09, *p* = 0.008; CDRSB, β = 0.10, *p* = 0.003; MMSE, β = −0.15, *p* < 0.001; dataset 2: ADAS13, β = 0.07, *p* = 0.004; CDRSB, β = 0.07, *p* = 0.005; MMSE, β = −0.09, *p* < 0.001) in longitudinal analysis. The variability of HFABP was associated with CSF P-tau (dataset 2: β = 3.62, *p* = 0.003). Structural equation modeling indicated that tau pathology mediated the relationship between HFABP and cognition.

**Conclusion:**

Our findings demonstrated that HFABP was significantly associated with longitudinal cognitive changes, which might be partially mediated by tau pathology.

## Introduction

Alzheimer’s disease (AD) is the primary cause of dementia in the elderly worldwide, and its prevalence is projected to triple over the next 20 years (Alzheimer’s Association, 2011; Prince et al., 2013). AD is characterized by neuropathological markers of neurofibrillary tangles made of filamentous hyperphosphorylated tau and neuritic amyloid-β plaques (Kirkitadze et al., 2002; Selkoe and Hardy, 2016). The underlying mechanisms of extensive and considerable neuropathology of AD are still unclear. Genome-wide association studies implicated lipid metabolism in several neurodegenerative diseases, including AD, and several studies demonstrated the association between lipid/lipoprotein metabolism and AD pathology (Hamilton et al., 2015; Marschallinger et al., 2020).

Changes in lipid metabolism during aging are crucial for a variety of biological processes (Papsdorf and Brunet, 2019). Dysfunction of lipid metabolism affects membrane lipid composition and fluidity, thus contributing to age-related neuronal cell dysfunction and neurologic disease (Mori et al., 2001; Siino et al., 2018). Therefore, lipid-binding proteins seem to be involved in the pathogenesis of AD. Recent studies suggested that heart fatty acid-binding protein (HFABP or FABP3), a lipid-binding protein facilitating the intracellular transport of fatty acids, may contribute to AD diagnosis and prognosis in the earliest stages (Rosén et al., 2011). A study found that the association between elevated levels of HFABP and longitudinal atrophy of crucial brain structures was significant among amyloid positive individuals and occurred irrespective of tau pathology (Chiasserini et al., 2017). However, other studies found that increased cerebrospinal fluid (CSF) HFABP was related to tau pathology and neurodegeneration (Chiasserini et al., 2010, 2017). There is a dearth of research investigating the longitudinal association of HFABP with cognition functioning and AD biomarkers and the mediational role of AD pathology in the relationship between lipid metabolism and cognition.

Here, in this investigation, we examined the association of AD biomarkers and cognition with HFABP from Alzheimer’s Disease Neuroimaging Initiative (ADNI) dataset. We evaluated whether CSF HFABP is associated with CSF AD biomarkers and cognition at baseline and follow-up and whether CSF HFABP change is associated with AD biomarkers and cognition over time. We also examined whether AD pathology is a potential mediator of the relationship between HFABP and cognition.

## Materials and methods

### Data description

The data used in the preparation of this were downloaded from the ADNI dataset^1^ (Weiner et al., 2010, 2012). The ADNI was launched in 2003 by the National Institute on Aging (NIA), the National Institute of Biomedical Imaging and Bioengineering (NIBIB), the Food and Drug Administration (FDA), private pharmaceutical companies, and non-profit organizations, as a $60 million, 5-year public-private partnership. For up-to-date information, see text footnote 1. Written consent was obtained at enrollment from all participants and the study was approved by each participating site’s institutional review board.

### Alzheimer’s disease neuroimaging initiative participants

Our study population consisted of all patients, including cognitively healthy control (CN), patients with mild cognitive impairment (MCI), and patients with AD dementia, with available CSF HFABP data from the ADNI cohort. Inclusion and exclusion criteria are described in detail online (see text footnote 1)

(Petersen et al., 2010). Finally, participants 55–90 years of age have been included, among whom individuals had available follow-up information. CN participants had a MMSE score of >24 and a clinical dementia rating score of 0. Patients with early MCI had a MMSE score of ≥24, a clinical dementia rating score of 0.5, preserved activities of daily living, and absence of dementia. Patients with AD dementia fulfilled the National Institute of Neurological Communicative Disorders and Stroke–Alzheimer Disease and Related Disorders Association criteria for probable AD (McKhann et al., 1984), had MMSE scores of 20–26, and had CDR scores of 0.5–1.0.

### Measurements of cerebrospinal fluid biomarkers analysis

We download the first data set of participants whose CSF HFABP protein levels were evaluated using a Myriad Rules Based Medicine platform (Human Discovery MAP, v1.0; see ADNI “Materials and methods” Section). A second data set from the Foundation for the National Institutes of Health (FNIH) Biomarker Consortium CSF Proteomics Project included 197 participants with CSF HFABP protein concentrations which were evaluated with the multiple Multi Reaction Monitoring (MRM) targeted mass spectroscopy at baseline and during follow-up.

Alzheimer’s Disease Neuroimaging Initiative CSF protocols, including Aβ1–42, tau, and p-tau181, have previously been described in detail (Shaw et al., 2009). The CSF Aβ1–42, tau, and p-tau181 levels were measured with the multiplex xMAP Luminex platform and Innogenetics INNO-BIA AlzBio3 (Innogenetics-Fujirebio, Ghent, Belgium) immunoassay reagents. The intra-assay coefficient of variation (CV) of duplicate determinations for concentration ranged from 2.5 to 5.9% for Aβ1–42, 2.2–6.3% for tau, and the inter-assay CV for CSF pool samples ranged from 5.1 to 14% for Aβ1–42, 2.7–11.2% for tau. Further information on standard operation procedures was described in previous publications (Shaw et al., 2009, 2011) and online (see text footnote 1).

### Cognitive measures

In ADNI, all participants received detailed cognitive evaluations, including the global cognition by Mini-Mental State Examination (MMSE), Clinical Dementia Rating sum of boxes (CDRSB), and the cognitive section of Alzheimer’s Disease Assessment Scale (ADAS13).

### Statistical analyses

All data management and analyses were conducted using R version 4.0.1 using the data. Table package version 1.14.0, the dplyr package version 1.0.2, the lme4 package version 1.1.29, the lmerTest package version 3.1.3, and the car package version 3.0.10. Figures were plotted using ggplot2 version 3.2.1. Additionally, stringr package version 1.4.0 and fst package version 0.9.4 were used for data management. All variables were log-transformed to improve normality.

First, multiple linear regression models were used to examine the cross-sectional relationship among CSF HFABP and cognition after adjusting for age, sex, education, and Apolipoprotein E (APOE) ε4 status, and AD core biomarkers after adjusting for age, sex, education, APOE ε4 status, and diagnosis. Second, to assess the association of baseline CSF HFABP levels with the longitudinal cognitive measures and AD core biomarkers. Linear mixed-effect models were used for the analysis, adjusting for the same covariables as the baseline models and additionally including time as an interacting variable with CSF HFABP levels. Third, estimated slopes for changes in the CSF HFABP concentrations were calculated for each individual using linear mixed-effect models for repeated measures. After that, the HFABP change rate was included in the linear mixed-effect models as independent variables to investigate the association between longitudinal CSF HFABP changes and longitudinal cognitive and AD core biomarkers changes. Finally, mediation models were conducted in the current study to investigate whether the association of HFABP with cognition was mediated by AD pathology. These models were adjusted for age, sex, education, and APOE ε4 status. Two other mediation analyses were used to determine whether the association of baseline and longitudinal CSF HFABP with longitudinal clinical outcomes were mediated respectively by baseline and longitudinal AD biomarkers. The same covariates as in the first model were used in two other models.

## Results

### Characteristics of participants

As for the ADNI dataset 1, 303 participants aged 56–89 years (mean age 75.14 years) were included in the present study at baseline. The mean education years of the study sample were 15.66, 39.4% were female, and 48.0% were APOE4 carriers (Table 1). As for ADNI dataset 2, 197 participants aged 55–89 years (mean age 72.69 years) were included in the present study at baseline. The mean education years of the study sample were 16.16, 44.4% were female, and 39.3% were APOE4 carriers (Table 1).

**TABLE 1:** Baseline characteristics of participants.

Characteristic	ADNI data 1	ADNI data 2
N	303	197
Age [mean (SD)]	75.14 (6.73)	72.69 (7.05)
Female (%)	129 (39.4)	87 (44.4)
Education [mean (SD)]	15.66 (3.04)	16.16 (2.81)
APOE carrier (%)	157 (48.0)	77 (39.3)
MMSE score [mean (SD)]	26.76 (2.56)	28.26 (1.86)
CDRSB score [mean (SD)]	1.71 (1.77)	0.88 (1.04)
ADAS13 score [mean (SD)]	18.54 (9.14)	13.05 (7.09)
**CSF AD biomarkers [mean (SD)]**		
Aβ42	905.26 (562.07)	1,162.69 (635.32)
Tau	307.76 (118.97)	281.17 (116.08)
P-tau	30.22 (13.63)	26.64 (13.07)

ADAS13, Alzheimer’s Disease Assessment Scale; ADNI, Alzheimer’s Disease Neuroimaging Initiative; CSF, cerebrospinal fluid; MMSE, Mini-Mental State Examination; CDRSB, clinical Dementia Rating sum of boxes; P-tau, phosphorylated tau; Aβ, Amyloid beta.

### The cross-section association of heart fatty acid-binding protein with cognition measures and Alzheimer’s disease biomarkers

**FIGURE 1:**
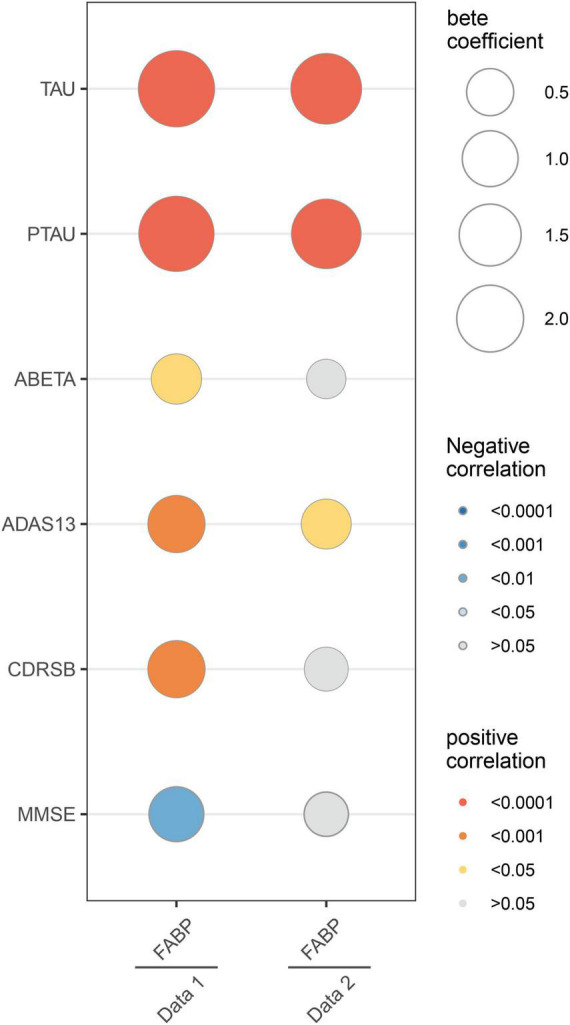
Linear associations between fatty acid binding protein and Mini-Mental State Examination (MMSE), Clinical Dementia Rating sum of boxes (CDRSB), ADAS13, Amyloid beta (ABETA), TAU, and PTAU. The size of the bubble represents the correlation levels. The color of the bubble represents the beta coefficient levels. Red: positive correlation. Blue: negative correlation. MMSE, Mini-Mental State Examination; CDRSB, Clinical Dementia Rating sum of boxes; ADAS13, the cognitive section of Alzheimer’s Disease Assessment Scale; ABETA, Amyloid beta; P-tau, phosphorylated tau.

In dataset 1, multivariate analyses showed the strong association of CSF HFABP with cognition measures and AD biomarkers. Individuals with higher HFABP levels had lower cognition measures, as indicated by higher ADAS13 score (β = 0.68, *p* < 0.001), higher CDRSB score (β = 0.69, *p* < 0.001), and lower MMSE score (β = −0.60, *p* = 0.002), and had higher concentration of P-tau (β = 2.04, *p* < 0.001). In dataset 2, similar results were observed in the relationship between HFABP and P-tau (β = 1.51, *p* < 0.001). However, there were no significant relationships between cognition measures, except ADAS13 score (β = 0.39, *p* = 0.021). This demonstrated the positive association of HFABP with P-tau and the negative association with cognition (Figure 1 and Supplementary Table 1).

### The longitudinal association of heart fatty acid-binding protein with cognition measures and Alzheimer’s disease biomarkers

**FIGURE 2:**
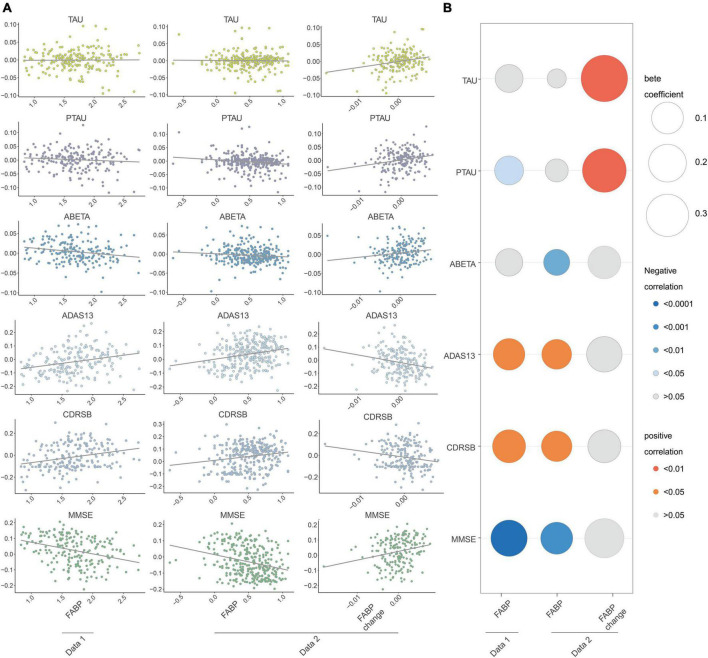
Longitudinal association between fatty acid binding protein and Mini-Mental State Examination (MMSE), Clinical Dementia Rating sum of boxes (CDRSB), ADAS13, Amyloid beta (ABETA), TAU, and PTAU. **(A)** Scatter plots of the association of heart fatty acid-binding protein (HFABP) and HFABP change with cognition and AD biomarkers changes; **(B)** standardized coefficients for the association of HFABP and HFABP change with cognitive measures and AD biomarkers. The size of the bubble represents the correlation levels. The color of the bubble represents the beta coefficient levels (interacted with time). Red: positive correlation. Blue: negative correlation. MMSE, Mini-Mental State Examination; CDRSB, Clinical Dementia Rating sum of boxes; ADAS13, the cognitive section of Alzheimer’s Disease Assessment Scale; ABETA, Amyloid beta; P-tau, phosphorylated tau.

To examine the association of baseline HFABP with cognitive measures and AD biomarkers, we constructed mixed effects models of these measures and biomarkers and observed that HFABP was associated with rate of cognitive change (interacted with time) in both datasets (dataset 1: ADAS13, β = 0.09, *p* = 0.008; CDRSB, β = 0.10, *p* = 0.003; MMSE, β = −0.15, *p* < 0.001; dataset 2: ADAS13, β = 0.07, *p* = 0.004; CDRSB, β = 0.07, *p* = 0.005; MMSE, β = −0.09, *p* < 0.001). However, the results for the association between HFABP and AD biomarkers were inconsistent of both dataset 1 and dataset 2. Baseline HFABP level was associated with P-tau (β = −0.06, *p* = 0.015) on dataset 1 and Aβ42 (β = −0.04, *p* = 0.008) on dataset 2 in a longitudinal model. To determine whether HFABP variability is associated with cognitive measures and AD biomarkers, we calculated the difference in CSF HFABP levels between baseline and follow-up and associated this difference with cognitive measures and AD biomarkers. HFABP variability was associated with increased CSF protein level of P-tau (β = 3.62, *p* = 0.003). This is consistent with the association between baseline HFABP and P-tau in cross-section and longitudinal models (Figure 2 and Supplementary Table 2).

### Causal mediation analyses

**FIGURE 3:**
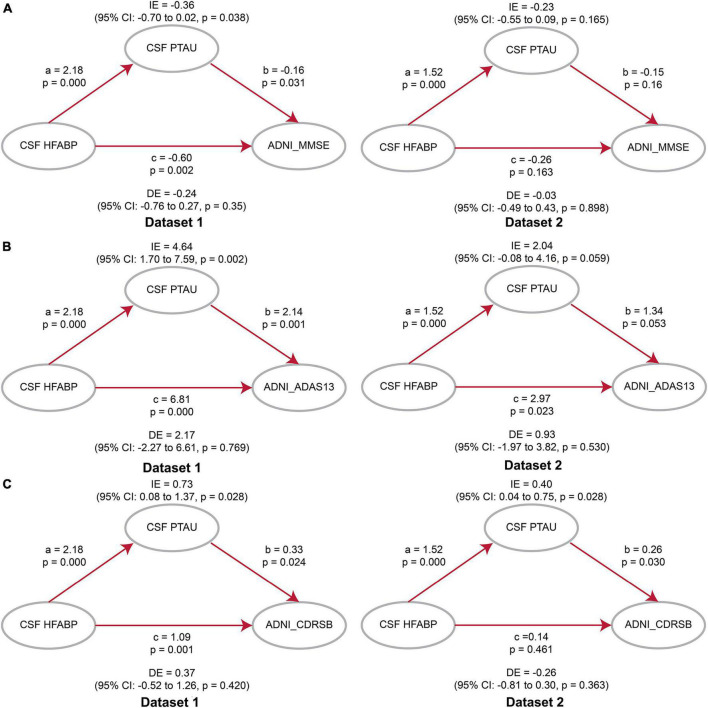
Mediation analyses showed that the relationship between heart fatty acid-binding protein (HFABP) and cognitive measures were mediated by tau pathology. **(A)** Global cognition measured by Mini-Mental State Examination (MMSE); **(B)** global cognition measured by ADAS13; **(C)** global cognition measured by CDRSB. MMSE, Mini-Mental State Examination; CDRSB, Clinical Dementia Rating sum of boxes; ADAS13, the cognitive section of Alzheimer’s Disease Assessment Scale; ABETA, Amyloid beta; P-tau, phosphorylated tau; IE, Indirect effects; DE, Direct effects.

**FIGURE 4:**
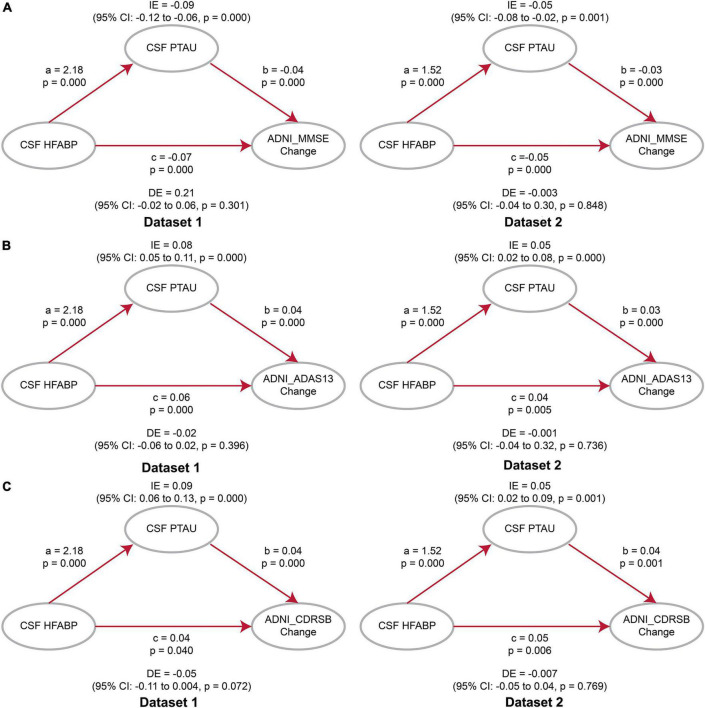
Mediation analyses showed that the relationship between heart fatty acid-binding protein (HFABP) and longitudinal cognition change was mediated by tau pathology. **(A)** Global cognition measured by Mini-Mental State Examination (MMSE); **(B)** global cognition measured by ADAS13; **(C)** global cognition measured by Clinical Dementia Rating sum of boxes (CDRSB). MMSE, Mini-Mental State Examination; CDRSB, Clinical Dementia Rating sum of boxes; ADAS13, the cognitive section of Alzheimer’s Disease Assessment Scale; ABETA, Amyloid beta; P-tau, phosphorylated tau; IE, Indirect effects; DE, Direct effects.

Considering the association of HFABP with cognition and tau pathology shown above, HFABP was associated with not only cognitive impairment but also CSF P-tau concentration. We investigate whether high HFABP levels contribute to cognitive impairment *via* tau pathology. In dataset 1, we found that the relationship between HFABP and cognition (MMSE: IE = −0.36, *p* = 0.038; ADAS13: IE = 4.64, *p* = 0.002; CDRSB: IE = 0.73, *p* = 0.028) and longitudinal cognition change (MMSE change: IE = −0.09, *p* = 0.000; ADAS13 change: IE = 0.08, *p* = 0.000; CDRSB change: IE = 0.09, *p* = 0.000) was mediated by tau pathology (Figures 3, 4 and Supplementary Tables 3, 4). However, in dataset 2, we only found tau pathology mediated the relationship between HFABP and longitudinal cognition change (MMSE change: IE = −0.05, *p* = 0.001; ADAS13 change: IE = 0.05, *p* = 0.000; CDRSB change: IE = 0.05, *p* = 0.001) (Figures 3, 4 and Supplementary Tables 3, 4).

## Discussion

The present study found that (1) HFABP was associated with baseline tau pathology; (2) HFABP was associated with longitudinal cognition change; (3) HFABP variability was associated with an increased CSF protein level of P-tau; and (4) the influence of HFABP on cognition was mediated by tau pathology. These findings consolidated the close relationships of HFABP with AD pathology and cognition, supporting the validity of the biomarker-based diagnosis of preclinical AD.

Previous studies found that CSF levels of HFABP in AD patients were higher than in MCI subjects and older people without cognition impairment (Chiasserini et al., 2017; Höglund et al., 2017; Dulewicz et al., 2021). Furthermore, other studies found significantly elevated CSF HFABP levels have been described in MCI subjects compared with the cognitively healthy group, but no difference between the dementia group and the progressive MCI subgroup (Guo et al., 2013). These results demonstrated that CSF levels of HFABP are already increased in the early stages of AD and increased with the progress of AD. This is in line with our studies, as we observed the association between baseline CSF HFABP and longitudinal cognition change. These findings suggested that changes in the CSF levels of HFABP may reflect the roles of lipid-related metabolism in the development of AD. High HFABP levels might result from increasing pathological processes and associate with the neurodegeneration process. Previous studies found that HFABP was significantly correlated with tau pathology (Chiasserini et al., 2010, 2017). The results of these studies are consistent with our research. We also found the longitudinal link between HFABP and tau pathology. Additionally, a study suggested a relationship between HFABP and neurodegeneration-related amyloid pathology and brain atrophy, while we failed to identify the association in the present study (Desikan et al., 2013).

The mechanisms underlying the association between HFABP and cognition remain unknown. Several possible mechanisms might be implicated. First, HFABP in the brain may regulate the lipids components of neuronal cell membranes, thereby affecting synaptic degeneration and regeneration and involving in a number of neurodegeneration diseases (Sellner et al., 1995; Mauch et al., 2001; Shioda et al., 2010; Yamamoto et al., 2018). Second, lipid rafts might mediate pathogenesis-related proteins, including α-synuclein (Fortin et al., 2004; Yabuki et al., 2020) and prions (Hooper, 2005; Taylor and Hooper, 2007), in a number of protein-misfolding neurodegenerative disorders. Thus, we speculated that HFABP and tau pathology have synergistic effects on AD development. Third, HFABP may also regulate dopamine D2R (dopamine receptor 2) function in the striatum and anterior cingulate cortex and mediates αSyn neurotoxicity in septal GABAergic neurons to affect cognitive function and emotional behavior (Yamamoto et al., 2018; Matsuo et al., 2021). Finally, recent studies suggested HFABP was associated with specific features of brain atrophy and white matter Hyperintensities burden, independently of amyloid and tau pathology biomarkers (Clark et al., 2022; Vidal-Piñeiro et al., 2022).

There are several limitations to this work. First, our study was limited by sample size. The sample size was too small for performing subgroup analyses to detect significant differences. Second, our study included patients with and without dementia to maximize the sample size, which might introduce heterogeneity and slight bias. Finally, we did not determine the plasma levels of HFABP, which will likely influence the CSF levels of HFABP.

## Conclusion

In summary, the present study demonstrated that HFABP was associated with tau pathology and longitudinal cognitive function. Tau pathology might partially mediate the association between HFABP and cognition. Further analyses in large cohorts are needed to validate such findings. Our findings suggested that tau pathology may mediate the role of lipid metabolism in AD development.

## Data availability statement

Publicly available datasets were analyzed in this study. This data can be found in the ADNI at http://adni.loni.usc.edu.

## Ethics statement

Ethical approval was obtained by the ADNI investigators. All participants provided written informed consent. The study conformed with the Declaration of Helsinki.

## Alzheimer’s disease neuroimaging initiative

The data used in this article were obtained from the Alzheimer’s Disease Neuroimaging Initiative (ADNI) database (adni.loni.usc.edu). As such, the investigators within the ADNI contributed to the design and implementation of ADNI and/or provided data but did not participate in the analysis or writing of this report. A complete listing of ADNI investigators can be found at: http://adni.loni.usc.edu/wpcontent/uploads/how_to_apply/ADNI_Acknowledgement_List.pdf.

## Author contributions

Z-TW and LT conceptualized the study and revised the manuscript. YF, L-YH, C-CT, and X-PC analyzed and interpreted the data, drafted and revised the manuscript, did the statistical analysis, and prepared all the figures. All authors contributed to wrote and revised the manuscript and approved the final version.

## Footnotes

1
https://adni.loni.usc.edu/
