# Supplementary material for: Heart fatty acid-binding protein is associated with phosphorylated tau and longitudinal cognitive changes
Source: Front Aging Neurosci. 2022 Oct 10;14:1008780. doi: 10.3389/fnagi.2022.1008780 (PMC9588952; doi:10.3389/fnagi.2022.1008780)
Supplement: Supplementary file 1 [file Data_Sheet_1.docx]

**Supplementary Materials**

[**Supplement Table 1: 2**](#_Toc108608593)

[**Supplement Table 2: 3**](#_Toc108608594)

[**Supplement Table 3: 4**](#_Toc108608595)

[**Supplement Table 4: 5**](#_Toc108608596)

| Dataset | MMSE | CDRSB | ADAS13 | ABETA | TAU | PTAU |
| --- | --- | --- | --- | --- | --- | --- |
| Data 1 | -0.60  (-0.96 to -0.23) | 0.69  (0.32 to 1.05) | 0.68  (0.32 to 1.04) | 0.403  (0.082 to 0.724) | 2.14  (1.855 to 2.425) | 2.036  (1.749 to 2.323) |
| Data 2 | -0.28  (-0.62 to 0.06) | 0.27  (-0.06 to 0.60) | 0.39  (0.06 to 0.72) | 0.237  (-0.059 to 0.532) | 1.609  (1.359 to 1.859) | 1.514  (1.261 to 1.767) |

Supplement Table 1: linear associations between fatty acid binding protein and MMSE, CDRSB, ADAS13, ABETA, TAU, PTAU.

Abbreviations: Mini-Mental State Examination (MMSE), Clinical Dementia Rating sum of boxes (CDRSB), and the cognitive section of Alzheimer’s Disease Assessment Scale (ADAS13), Amyloid beta (ABETA), phosphorylated tau (P-tau).

| Dataset | Variable | MMSE | CDRSB | ADAS13 | ABETA | TAU | PTAU |
| --- | --- | --- | --- | --- | --- | --- | --- |
| Data 1 | FABP | -0.536  (-0.84 to -0.232) | 0.678  (0.355 to 1.001) | 0.749  (0.43 to 1.068) | 0.412  (0.096 to 0.728) | 2.091  (1.806 to 2.376) | 2.014  (1.726 to 2.303) |
|  | TIME | -0.102  (-0.141 to -0.063) | 0.135  (0.097 to 0.172) | 0.128  (0.094 to 0.162) | -0.021  (-0.047 to 0.005) | 0.057  (0.03 to 0.084) | 0.036  (0.012 to 0.06) |
|  | FABP: TIME | -0.151  (-0.223 to -0.08) | 0.103  (0.036 to 0.171) | 0.085  (0.023 to 0.147) | -0.038  (-0.088 to 0.011) | -0.036  (-0.086 to 0.015) | -0.044  (-0.088 to 0.001) |
| Data 2 | FABP | -0.162  (-0.419 to 0.095) | 0.157  (-0.128 to 0.443) | 0.33  (0.046 to 0.614) | 0.204  (-0.088 to 0.496) | 1.565  (1.314 to 1.816) | 1.506  (1.252 to 1.761) |
|  | TIME | 0.062  (-0.019 to 0.144) | -0.043  (-0.13 to 0.044) | -0.025  (-0.103 to 0.053) | 0.032  (-0.014 to 0.078) | 0.064  (0.019 to 0.109) | 0.065  (0.024 to 0.107) |
|  | FABP: TIME | -0.091  (-0.139 to -0.044) | 0.074  (0.023 to 0.124) | 0.066  (0.021 to 0.111) | -0.036  (-0.062 to -0.009) | -0.013  (-0.039 to 0.013) | -0.019  (-0.043 to 0.004) |
| Data 2 | FABP_CHANGE | 1.017  (-0.241 to 2.275) | -0.837  (-2.209 to 0.535) | -1.016  (-2.412 to 0.381) | 0.015  (-1.453 to 1.483) | -0.491  (-2.152 to 1.171) | -0.514  (-2.162 to 1.134) |
|  | TIME | -0.094  (-0.115 to -0.074) | 0.076  (0.055 to 0.098) | 0.09  (0.07 to 0.109) | -0.03  (-0.041 to -0.019) | 0.037  (0.027 to 0.048) | 0.028  (0.018 to 0.037) |
|  | FABP_CHANGE: TIME | 0.195  (-0.074 to 0.463) | 0.101  (-0.169 to 0.371) | -0.136  (-0.383 to 0.111) | 0.097  (-0.057 to 0.25) | 0.358  (0.22 to 0.495) | 0.294  (0.169 to 0.418) |

Supplement Table 2: Linear Mixed Effects Model between fatty acid binding protein and MMSE, CDRSB, ADAS13, ABETA, TAU, PTAU.

Abbreviations: Mini-Mental State Examination (MMSE), Clinical Dementia Rating sum of boxes (CDRSB), and the cognitive section of Alzheimer’s Disease Assessment Scale (ADAS13), Amyloid beta (ABETA), phosphorylated tau (P-tau), Fatty acid binding protein (FABP)

Supplement Table 3: Causal mediation modals of PTAU in relationship between fatty acid binding protein and MMSE.

| Data1: MMSE ~ FABP+PTAU | | | | |
| --- | --- | --- | --- | --- |
|  | Estimate | SE | p-value |  |
| ACME | -0.358 | 0.174 | 0.039 | * |
| ADE | -0.244 | 0.265 | 0.358 |  |
| Total Effect | -0.602 | 0.198 | 0.002 | ** |
| Data2: MMSE ~ FABP+PTAU | | | | |
|  | Estimate | SE | p-value |  |
| ACME | -0.734 | 0.323 | 0.023 | * |
| ADE | 0.280 | 0.396 | 0.480 |  |
| Total Effect | -0.454 | 0.361 | 0.208 |  |
| Data1: ADAS13 ~ FABP+PTAU | | | | |
|  | Estimate | SE | p-value |  |
| ACME | 5.990 | 1.330 | 0.000 | *** |
| ADE | 2.944 | 2.126 | 0.166 |  |
| Total Effect | 8.934 | 1.698 | 0.000 | *** |
| Data2: ADAS13 ~ FABP+PTAU | | | | |
|  | Estimate | SE | p-value |  |
| ACME | -0.734 | 0.323 | 0.023 | * |
| ADE | 0.280 | 0.396 | 0.480 |  |
| Total Effect | -0.454 | 0.361 | 0.208 |  |
| Data1: CDRSB ~ FABP+PTAU | | | | |
|  | Estimate | SE | p-value |  |
| ACME | 1.308 | 1.330 | 0.000 | *** |
| ADE | 0.075 | 0.388 | 0.847 |  |
| Total Effect | 1.383 | 0.330 | 0.000 | *** |
| Data2: CDRSB ~ FABP+PTAU | | | | |
|  | Estimate | SE | p-value |  |
| ACME | -0.734 | 0.323 | 0.023 | * |
| ADE | 0.280 | 0.396 | 0.480 |  |
| Total Effect | -0.454 | 0.361 | 0.208 |  |

Abbreviations: Mini-Mental State Examination (MMSE), Clinical Dementia Rating sum of boxes (CDRSB), and the cognitive section of Alzheimer’s Disease Assessment Scale (ADAS13), phosphorylated tau (P-tau), Fatty acid binding protein (FABP), Average causal mediation effects (ACME), average direct effects (ADE).

Supplement Table 4: Causal mediation modals of PTAU in relationship between fatty acid binding protein and MMSE change.

| Data1: MMSE change ~ FABP+PTAU | | | | |
| --- | --- | --- | --- | --- |
|  | Estimate | SE | p-value |  |
| ACME | -0.093 | 0.015 | 0.000 | *** |
| ADE | 0.021 | 0.020 | 0.303 |  |
| Total Effect | -0.072 | 0.017 | 0.000 | *** |
| Data2: MMSE change ~ FABP+PTAU | | | | |
|  | Estimate | SE | p-value |  |
| ACME | -0.039 | 0.014 | 0.006 | ** |
| ADE | -0.014 | 0.017 | 0.396 |  |
| Total Effect | -0.053 | 0.013 | 0.000 | *** |
| Data1: ADAS13 change ~ FABP+PTAU | | | | |
|  | Estimate | SE | p-value |  |
| ACME | 0.113 | 0.016 | 0.000 | *** |
| ADE | -0.046 | 0.020 | 0.022 | * |
| Total Effect | 0.067 | 0.016 | 0.000 | *** |
| Data2: ADAS13 change ~ FABP+PTAU | | | | |
|  | Estimate | SE | p-value |  |
| ACME | -0.039 | 0.014 | 0.006 | ** |
| ADE | -0.014 | 0.017 | 0.396 |  |
| Total Effect | -0.053 | 0.013 | 0.000 | *** |
| Data1: CDRSB change ~ FABP+PTAU | | | | |
|  | Estimate | SE | p-value |  |
| ACME | 0.106 | 0.017 | 0.000 | *** |
| ADE | -0.048 | 0.025 | 0.054 |  |
| Total Effect | 0.059 | 0.019 | 0.002 | ** |
| Data2: CDRSB change ~ FABP+PTAU | | | | |
|  | Estimate | SE | p-value |  |
| ACME | -0.039 | 0.014 | 0.006 | ** |
| ADE | -0.014 | 0.017 | 0.396 |  |
| Total Effect | -0.053 | 0.013 | 0.000 | *** |

Abbreviations: Mini-Mental State Examination (MMSE), Clinical Dementia Rating sum of boxes (CDRSB), and the cognitive section of Alzheimer’s Disease Assessment Scale (ADAS13), phosphorylated tau (P-tau), Fatty acid binding protein (FABP), Average causal mediation effects (ACME), average direct effects (ADE).
